# Human and Mouse CD8^+^CD25^+^FOXP3^+^ Regulatory T Cells at Steady State and during Interleukin-2 Therapy

**DOI:** 10.3389/fimmu.2015.00171

**Published:** 2015-04-15

**Authors:** Guillaume Churlaud, Fabien Pitoiset, Fadi Jebbawi, Roberta Lorenzon, Bertrand Bellier, Michelle Rosenzwajg, David Klatzmann

**Affiliations:** ^1^Department of Inflammation-Immunopathology-Biotherapy (I2B), Clinical Investigation Center for Biotherapies (CIC-BTi), Hôpital Pitié-Salpêtrière, Assistance Publique Hôpitaux de Paris (AP-HP), Paris, France; ^2^UMRS 959, Immunology-Immunopathology-Immunotherapy (I3), Sorbonne Université, Université Pierre-et-Marie-Curie, Institut national de la santé et de la recherche médicale (INSERM), Paris, France; ^3^FRE 3632, Immunology-Immunopathology-Immunotherapy (I3), Centre national de la recherche scientifique (CNRS), Paris, France

**Keywords:** immunological tolerance, immunotherapy, autoimmunity, T cell biology, immune response

## Abstract

In addition to CD4^+^ regulatory T cells (Tregs), CD8^+^ suppressor T cells are emerging as an important subset of regulatory T cells. Diverse populations of CD8^+^ T cells with suppressive activities have been described. Among them, a small population of CD8^+^CD25^+^FOXP3^+^ T cells is found both in mice and humans. In contrast to thymic-derived CD4^+^CD25^+^FOXP3^+^ Tregs, their origin and their role in the pathophysiology of autoimmune diseases (AIDs) are less understood. We report here the number, phenotype, and function of CD8^+^ Tregs cells in mice and humans, at the steady state and in response to low-dose interleukin-2 (IL-2). CD8^+^ Tregs represent approximately 0.4 and 0.1% of peripheral blood T cells in healthy humans and mice, respectively. In mice, their frequencies are quite similar in lymph nodes (LNs) and the spleen, but two to threefold higher in Peyer patches and mesenteric LNs. CD8^+^ Tregs express low levels of CD127. CD8^+^ Tregs express more activation or proliferation markers such as CTLA-4, ICOS, and Ki-67 than other CD8^+^ T cells. *In vitro*, they suppress effector T cell proliferation as well as or even better than CD4^+^ Tregs. Owing to constitutive expression of CD25, CD8^+^ Tregs are 20- to 40-fold more sensitive to *in vitro* IL-2 stimulation than CD8^+^ effector T cells, but 2–4 times less than CD4^+^ Tregs. Nevertheless, low-dose IL-2 dramatically expands and activates CD8^+^ Tregs even more than CD4^+^ Tregs, in mice and humans. Further studies are warranted to fully appreciate the clinical relevance of CD8^+^ Tregs in AIDs and the efficacy of IL-2 treatment.

## Introduction

T cell development in the thymus comprises the positive selection of functional T cells capable of supporting adaptive immunity and the elimination of highly self-reactive T cells. The latter process is leaky and some autoreactive effector T cells (Teffs) escape into the periphery where they are regulated by peripheral tolerance mechanisms. In healthy individuals, multiple cell subsets with immunoregulatory properties, among CD4^+^ and CD8^+^ T cell or B cell populations, control these potentially harmful Teffs (1–3).

The best characterized of such regulatory cell populations are the natural CD4^+^CD25^+^FOXP3^+^ thymic-derived regulatory T cells (CD4^+^ Tregs). Their major role in the maintenance of immunological self-tolerance and immune homeostasis (4, 5) is illustrated by the rapid development of autoimmune diseases (AIDs) in normal mice upon their depletion (4) and also by the occurrence of severe AID, allergy, and immunopathology in humans with a mutated FOXP3 gene (6). CD4^+^ Treg biology has dominated research on regulatory cells in AIDs. More recently, other regulatory cell populations have received attention, and notably CD8^+^ suppressor cells (CD8^+^ Tsups) for which evidence that they are involved in AIDs is growing (7–9). However, CD8^+^ Tsups are less well characterized than CD4^+^ Tregs.

CD8^+^ Tsups are functionally defined populations of CD8^+^ T cells endowed with immunosuppressive functions (10–14). Several subpopulations of CD8^+^ Tsups have been described based on the expression of CD8αα, CD25, CD38, CD45RA, CD45RO, CD56, FOXP3, CXCR3, LAG-3, CD103, CD122, and/or HLA-G, as well as the absence of CD28 and CD127 (2, 7, 8, 15). The different CD8^+^ Tsup subsets are multiply involved in the pathophysiology of different AIDs (16). Their suppressive activity in AIDs was first demonstrated in CD8-depleted mice, which were more susceptible to a second induction of experimental autoimmune encephalomyelitis (17). CD8-deficient mice were also more susceptible to relapse of autoimmune arthritis after immunization with self-antigens (18). In non-obese diabetic mice, antigen-specific CD8^+^ Tsups were able to not only prevent but also reverse type 1 diabetes (15). Notably, in patients with systemic lupus erythematosus (19–21), inflammatory bowel disease (22), or multiple sclerosis (23–26) defective functions and/or reduced numbers of CD8^+^ Tsups have been reported. CD8^+^ Tsup biology is also less well known than that of CD4^+^ Tregs, due in part to their small numbers, which render functional studies difficult.

Only some CD8^+^ Tsups express FOXP3, the master regulator of CD4^+^ Treg differentiation and function, as well as CD25, and will be referred to here as CD8^+^ Tregs. Many groups are now suggesting that FOXP3 expression might represent a good indicator of a bona fide suppressive function (7, 27–30). Notably, it was demonstrated in lupus-prone mice that silencing FOXP3 with siRNA abrogates the ability of CD8^+^ Tregs to suppress anti-DNA antibodies (27, 29).

As CD8^+^ Tregs express CD25, the question of their sensitivity to interleukin-2 (IL-2) is relevant. Stimulation with IL-2 is indeed crucial for CD4^+^ Tregs. Mice deficient in IL-2 or in IL-2 receptor develop systemic AIDs that have been related to impaired development, survival, and function of CD4^+^ Treg cells (1, 31). However, the lack of IL-2 signaling effects on CD8^+^ Tregs has not been specifically evaluated in these models. Nevertheless, it is already known that, like CD4^+^ Tregs, CD8^+^ Tregs expand in response to IL-2 treatment, as we previously showed in a clinical trial using low-dose IL-2 in hepatitis C virus-induced vasculitis (32) and type 1 diabetes (33).

We sought to characterize CD8^+^CD25^+^FOXP3^+^ Tregs in mice and humans, phenotypically and functionally, at steady state and under IL-2 stimulation *in vitro* and *in vivo*. We show here that CD8^+^ Tregs are highly suppressive and responsive to IL-2. Our results warrant further study of these bona fide CD8^+^ Tregs in AID pathophysiology and therapy.

## Materials and Methods

### Human blood samples

Blood samples from healthy volunteers were obtained from the Etablissement français du sang (EFS) at the Pitié-Salpêtrière Hospital in Paris, France. Informed consent was obtained from each volunteer.

Blood samples from type 1 diabetes patients were obtained from the DF-IL2 trial (clinicaltrials.gov identifier NCT01353833). Patients were treated at the Centre d’Investigation Clinique (CIC)-Paris Est of la Pitié-Salpêtrière Hospital in Paris, France. Details about the clinical trial and the patients’ clinical characteristics are described in Hartemann et al. (34). Written informed consent was obtained from all participants before enrolment in the study. The study was approved by the institutional review board of the Pitié-Salpêtrière Hospital, and was done in accordance with the Declaration of Helsinki and good clinical practice guidelines.

### Mice

BALB/cJRj and C57Bl/6 mice were from Janvier. Transgenic C57Bl/6 FOXP3-GFP mice that express green fluorescent protein (GFP) in FOXP3^+^ cells were kindly provided by Dr. Malissen of the Centre d’immunologie de Marseille Luminy (France). CD3 KO mice were from CDTA of Orléans (France). Animals were maintained in our animal facility under specific pathogen-free conditions in agreement with current European legislation on animal care, housing, and scientific experimentation. All procedures were approved by the Regional Ethics Committee on Animal Experimentation No. 5 of the Ile-de-France region (Ce5/2012/031).

#### Preparation of tissue-infiltrating lymphocytes in mice

Spleen, lymph nodes (LNs) (mesenteric LN, MLN; cervical LN, CLN; pancreatic LN, PaLN), and Peyer’s patches (PP) were isolated and dissociated in PBS 3% fetal calf serum (FCS). Pancreas was digested with collagenase/DNase solution in RPMI medium, and filtered as described (35). A Ficoll (Sigma-Aldrich) gradient was used to isolate tissue-infiltrating lymphocytes.

#### Interleukin-2 treatment in mice

Eight-week-old female BALB/c mice received intraperitoneal injections of 50,000 or 100,000 IU of recombinant human IL-2 (Proleukin, Novartis) or PBS daily for 5 days. Twenty-four hours after the last injection, blood was collected and analyzed by flow cytometry.

### Flow cytometry

#### Analysis of cell surface and intracellular markers and FOXP3 expression in mice

Fresh total cells from the respective tissues were directly stained with the following monoclonal antibodies (mAbs) at predetermined optimal dilutions for 20 min at 4°C: CD3-PE, CD8-Alexa700, CD4-HorizonV500, CD127-FITC, and CD25-PeCy7 (eBioscience). Intracellular detection of FOXP3 (FOXP3-E450, eBioscience), CTLA-4 (CTLA-4-APC, eBioscience), Ki-67 (Ki-67-FITC, BD Pharmingen), and Bcl-2 (Bcl-2-PE, BD Pharmingen) was performed on fixed and permeabilized cells using appropriate buffer (eBioscience) (incubation 30 min at 4°C). Cells were acquired on an LSR II flow cytometer (Becton Dickinson) and analyzed using FlowJo software (Tree Star, Inc.). Dead cells were excluded by forward/side scatter gating.

#### Analysis of cell surface markers and FOXP3 expression in humans

Direct *ex vivo* immunostaining was performed on 50 μL of lithium heparinized fresh whole blood from healthy donors using the PerFix-nc kit (Beckman Coulter) according to the manufacturer’s instructions. Briefly, 5 μL of fixative reagent was added to the blood for 15 min at room temperature in the dark, before adding antibodies diluted in 300 μL of permeabilizing reagent for 1 h at room temperature in the dark. Staining was performed with FOXP3^−^ AF647 (clone 259D), CD25-PE, CD127-PE-Cy7, CD8-Chrome Orange, CD4-Pacific Blue, CD3-FITC, CD103-APC, ICOS-FITC, CD122-FITC, and CCR7-PE mAbs, all from Beckman Coulter. CTLA-4-PeCy7 mAb was from Biolegend. Samples were acquired on a Navios cytometer (Beckman Coulter) and analyses were performed using Kaluza software (Beckman Coulter). Matched mouse isotype control antibodies were used.

Instrument settings (gain, compensation, and threshold) were set with machine software (Navios Software; Beckman Coulter) in conjunction with calibration beads (Flow-set beads, Cytocomp kit, and CYTO-TROL Control Cells). Machine reproducibility was verified with standardized beads (Flow-check).

In mice and in humans, CD4^+^ Tregs were defined as CD25^+^ FOXP3^+^ cells among CD4^+^ T cells, and effector CD4^+^ T cells as FOXP3^−^ cells among CD4^+^ T cells. CD8^+^ Tregs were defined as CD25^+^FOXP3^+^ cells among CD8^+^ T cells, and effector CD8^+^ T cells as FOXP3^−^ cells among CD8^+^ T cells.

#### Absolute numbers of CD8^+^ Tregs and CD4^+^ Tregs in peripheral blood

Briefly, PBMC subsets (CD4^+^ Tregs, CD8^+^ Tregs) counts (cells/μL) were established from fresh blood samples using Flowcount fluorescents beads (Beckman Coulter) as internal standard (33).

#### pSTAT5 staining procedure

The pSTAT5 staining was assessed using PerFix EXPOSE reagents from Beckman Coulter as previously described (36). Briefly, fresh lithium heparinized whole blood was stained using anti-CD4, anti-CD25, anti-FOXP3, and PE-conjugated anti-phosphorylated STAT5 (Beckman Coulter) antibodies. Blood samples were stimulated with increasing hIL-2 (proleukin, Novartis) concentrations at 37°C for 10 min. Cell surface staining was then performed. Whole blood was incubated for 5 min (37°C, incubator). Samples were fixed for 10 min at room temperature in the dark, using 50 μL of fixative reagent (PerFix EXPOSE). Aliquots were permeabilized using 1 mL of permeabilizing reagent (PerFix EXPOSE), and incubated for 5 min at 37°C. Samples were centrifuged at 300 × *g* for 5 min, and the supernatant was completely discarded by aspiration. Then, intracellular staining with a mixture of 100 μL of staining reagent including PE-anti-pSTAT5 and AF647-anti-FOXP3 antibodies was performed for 30 min (room temperature) and cells were washed with 3 mL of washing buffer (PerFix EXPOSE). The supernatant was completely discarded by aspiration and 300 μL of PBS was added.

### *In vitro* suppression assay

Four C57Bl/6 FOXP3-GFP mice were sacrificed, and spleen and LN were collected and dissociated in PBS 3% FCS. Cells were stained with CD3, CD4, CD8, and CD25 (as described above) and then sorted using a FACS ARIA cell sorter (Becton Dickinson). CD4^+^GFP^−^ (CD4^+^ Teffs), CD4^+^CD25^+^GFP^+^ (CD4^+^ Tregs), and CD8^+^CD25^+^GFP^+^ (CD8^+^ Tregs) were collected. The purity of cell preparations exceeded 97%. CD4^+^ Teffs were cultured in RPMI 1640 medium supplemented with 5% FCS, 2 mmol/L l-glutamine, 100 U/mg/mL penicillin/streptomycin at 5 × 10^4^ cells/well, and variable numbers of regulatory cells were added in the presence of 7.5 × 10^4^ total splenocytes from CD3 KO mice and anti-CD3 (final concentration 0.1 μg/mL, BioXell).

3H-thymidine (1 μCi/well) incorporation was evaluated during the final 16 h of the 3-day culture.

### Statistical analyses

Statistical significance was evaluated using GraphPad Prism version 5.00 for Windows (GraphPad Software, San Diego, CA, USA, http://www.graphpad.com) and calculated using the paired *t*-test, Mann–Whitney test (comparison of means, unpaired test, non-parametric test, two-tail *p* value), or one-way ANOVA test, with *p* < 0.05 (*) taken as statistical significance (**p < 0.01, ***p < 0.001, NS, non-significant).

## Results

### Characterization and phenotype of CD8^+^CD25^+^FOXP3^+^ Tregs

**Figure 1:**
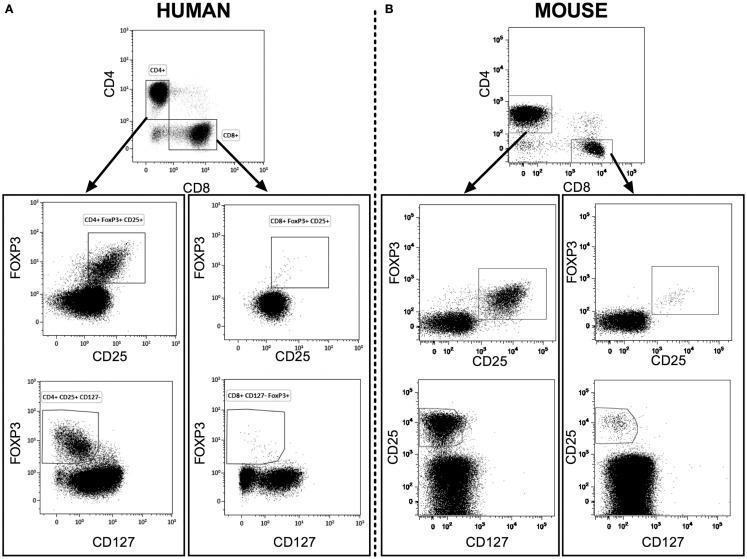
**Representative flow cytometry analysis of CD4^+^ Tregs and CD8^+^ Tregs from fresh heparinized peripheral blood of human healthy donors [human (A)] and of naive BALB/c mice [mouse (B)]**. CD4^+^ and CD8^+^ T cells were gated from CD3^+^ lymphocytes.

We measured the percentages and absolute numbers of CD8^+^ Tregs and CD4^+^ Tregs in human blood and in blood and lymphoid organs from C57Bl/6 and BALB/c mice. A representative gating strategy for CD8^+^CD25^+^FOXP3^+^ Treg immunophenotyping by flow cytometry in humans (Figure 1A) and in BALB/c mice (Figure 1B) is shown in Figure 1. In mice, CD4^+^ Tregs and CD8^+^ Tregs were solely defined by the co-expression of FOXP3 and CD25. In human and to a lesser extent in mouse, CD4^+^ Tregs and CD8^+^ Tregs were also characterized by low levels of CD127 compared to Teffs (Figure S1 in Supplementary Material).

**Figure 2:**
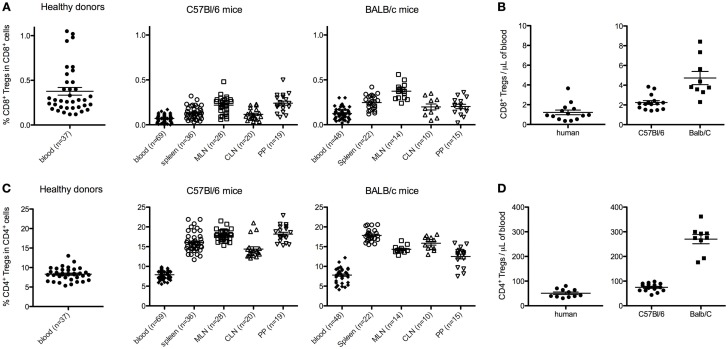
**Percentages and absolute counts of CD8^+^ Tregs among CD8^+^ T cells [respectively (A,B)], and of CD4^+^ Tregs among CD4^+^ T cells [respectively (C,D)] in human healthy donors and C57Bl/6 or BALB/c naive mice**. Human data are from 10 to 37 healthy blood donors. Mouse data are from a pool of at least three different experiments analyzing 8- to 12-week-old female mice. Individual values and mean ± SEM are shown. MLN, mesenteric lymph nodes; CLN, cervical lymph nodes; PP, Peyer’s patches.

In peripheral blood mononuclear cells (PBMCs) from human healthy donors, the percentage of CD8^+^ Tregs among CD8^+^ T cells was variable and below 1% (0.38 ± 0.26, mean ± SD, *n* = 37; Figure 2A). It is noteworthy that a wide range of values was observed (0.1–1%) with a quite heterogeneous distribution, since few individuals had markedly higher values (Figure 2A). In comparison, the percentage of CD4^+^ Tregs among CD4^+^ T cells in human blood was higher and less variable (8.3 ± 1.6) (Figure 2C).

In mice, the percentages of CD8^+^ Tregs among CD8^+^ T cells were 0.07 ± 0.04 and 0.12 ± 0.07% in peripheral blood of C57Bl/6 and BALB/c (Figure 2A). Similar or slightly higher values were observed in spleen and CLN in both mouse strains. When MLN and PP were analyzed, two to threefold higher values were observed as compared with blood values, except for PP in BALB/c mice. In comparison, in these different lymphoid tissues, the percentages of CD4^+^ Tregs among CD4^+^ T cells were around 50-fold higher in both C57Bl/6 and BALB/c mice (Figure 2C).

These 50-fold differences in percentages of CD4^+^ Tregs and CD8^+^ Tregs were also observed when looking at absolute numbers (Figures 2B,D) in both human and mouse.

**Figure 3:**
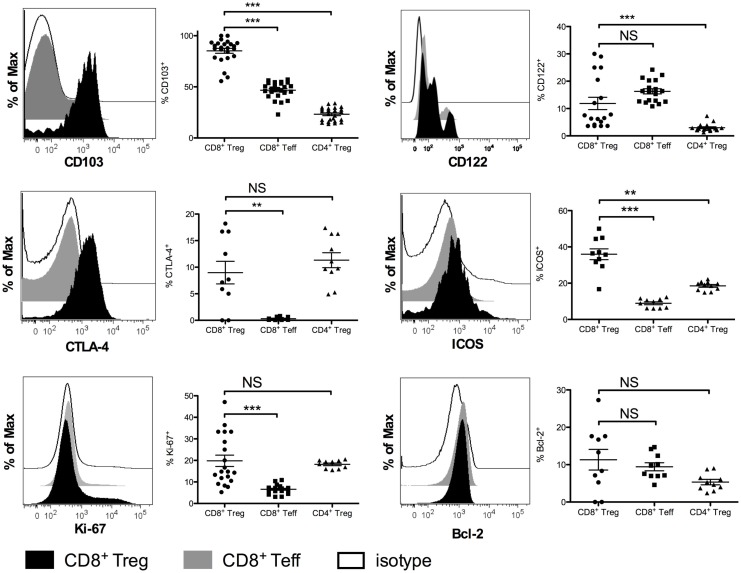
**Phenotypic characterization of CD8^+^CD25^+^FOXP3^+^ Tregs (CD8^+^ Tregs) compared with CD8^+^CD25^−^FOXP3^−^effector CD8^+^ T cells (CD8^+^ Teffs) and with CD4^+^CD25^+^FOXP3^+^ Tregs (CD4^+^ Tregs) in female BALB/c mice**. Representative histograms showing expression of CD103, CTLA-4, Ki-67, CD122, ICOS, and Bcl-2 in CD8^+^ Teffs (gray) and in CD8^+^ Tregs (black) compared to isotype control (white) are shown. Corresponding percentages in the overall population are shown. Individual values and mean ± SEM are shown.

We compared the phenotype of mouse CD8^+^ Tregs with that of other CD8^+^ T cells and of CD4^+^ T cells, focusing mainly on proteins associated with the regulatory function of CD4^+^ Tregs (Figure 3). Most CD8^+^ Tregs expressed CD103 (85.2 ± 11.8%) and some were CD122^+^ (11.9 ± 9.6%) with significantly higher levels compared with CD8^+^ Teffs only for CD103 expression (*p* < 0.0001). Approximately 36% of them were ICOS^+^ and only 9% expressed more CTLA-4 than CD8^+^ Teffs (*p* < 0.0001 and *p* = 0.0025, respectively). CD8^+^ Tregs had a proliferation rate measured by expression of Ki-67 (19.8 ± 11.7%) that was more than three times higher than that of CD8^+^ Teffs (6.6 ± 2.0%, *p* < 0.0001), whereas anti-apoptotic Bcl-2 marker expression was the same in CD8^+^ Tregs and CD8^+^ Teffs. This phenotype was similar in CD4^+^ Tregs, except for a lower expression of CD103, CD122, and ICOS (Figure 3).

A large fraction of human CD8^+^ Tregs express CTLA-4 (59 ± 19%) (Figure S2 in Supplementary Material) as do CD4^+^ Tregs. In contrast, CD8^+^ Tregs do not express any of the other markers we tested, i.e., CD103, CD122, ICOS, and CCR7 (data not shown).

### Suppressive activity of CD4^+^ Tregs and CD8^+^ Tregs

**Figure 4:**
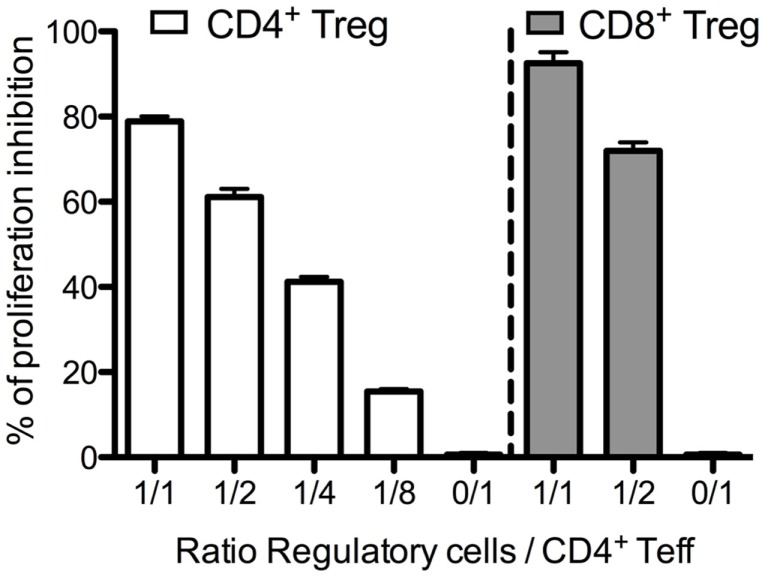
**Suppressive activity of murine CD4^+^ Tregs and CD8^+^ Tregs**. Freshly sorted CD4^+^GFP^−^ (CD4^+^ Teffs) cells were cultured with CD4^+^ Tregs or CD8^+^ Tregs at various ratios for 3 days, and tritiated thymidine was added for the last 16 h of the culture. Proliferation was assessed by tritiated thymidine incorporation measurement.

We next evaluated the suppressive capacity of CD8^+^ Tregs. For that purpose, we used C57Bl/6 FOXP3-GFP mice. In these knock-in mice, GFP is expressed only in *FOXP3* positive cells and can thus be used as a marker for flow cytometry sorting of viable CD8^+^ Tregs. CD8^+^ Tregs (CD8^+^CD25^+^GFP^+^) and CD4^+^ Tregs (CD4^+^CD25^+^GFP^+^) were sorted and evaluated for their capacity to suppress CD4^+^ Teffs (CD4^+^GFP^−^ cells). CD8^+^ Tregs appeared slightly more suppressive than CD4^+^ Tregs (Figure 4). At a 1:1 Treg:Teff ratio, 95% of CD4^+^ Teff suppression was observed with CD8^+^ Tregs and 77% with CD4^+^ Tregs. Similar differences were observed at a 1:2 ratio in the same experiment and similar results were also obtained with human CD8^+^ Tregs (28).

### *In vitro* sensitivity to IL-2 of CD4^+^ Tregs and CD8^+^ Tregs

**Figure 5:**
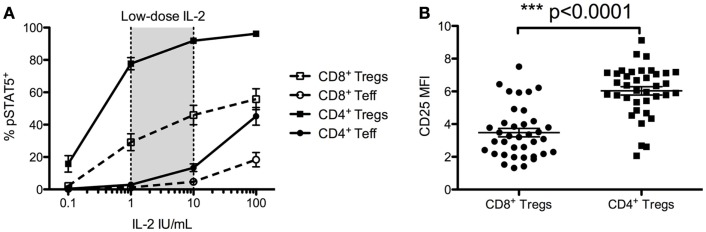
**Foxp3^+^ CD25^high^ CD127^low^ CD4^+^ T cells (CD4^+^ Tregs) and Foxp3^+^ CD25^high^ CD127^low^ CD8^+^ T cells (CD8^+^ Tregs) responsiveness to IL-2 and their comparative CD25 cell surface expressions**. **(A)** Total fresh blood from healthy donors was *ex vivo* stimulated with different doses of IL-2 for 10 min, and STAT5 phosphorylation was measured on different T cell subsets. Results are expressed in mean ± SE, *n* = 7–22. Low-dose IL-2 has been defined as the dose inducing high STAT5 phosporylation on CD4^+^ Tregs but not on CD4^+^ Teff. **(B)** Mean fluorescence intensity (MFI) of CD25 on CD4^+^ Tregs and CD8^+^ Tregs (*n* = 37) from fresh unstimulated whole blood from healthy donors. Individual values and mean ± SEM are shown.

As CD4^+^ Tregs and CD8^+^ Tregs constitutively express CD25, included in the high-affinity IL-2 receptor, we investigated their sensitivity to *in vitro* IL-2 activation. We evaluated the IL-2-induced phosphorylation of STAT5 proteins (pSTAT5) by flow cytometry (Figure 5). CD4^+^ Tregs are exquisitely sensitive to IL-2 activation, activated with doses 20- to 40-fold lower than those required to activate CD4^+^ Teffs. It is noteworthy that CD8^+^ Tregs had the same 20- to 40-fold higher sensitivity to IL-2 activation as CD8^+^ Teffs, but were 2- to 4-fold less sensitive than CD4^+^ Tregs (Figure 5A). At 1 IU/mL, the proportion of pSTAT5 positive cells was of 77.7 ± 3.8% in CD4^+^ Tregs, 29.1 ± 5.2% in CD8^+^ Tregs, but only 2.9 ± 0.5 and 1.2 ± 0.3% in CD4^+^ and CD8^+^ Teffs, respectively. At 10 IU/mL, these proportions were 91.9 ± 1.5% for CD4^+^ Tregs, 46.0 ± 6.0% for CD8^+^ Tregs, and only 13.4 ± 2.4% for CD4^+^ Teffs and 4.7 ± 1.7% for CD8^+^ Teffs. These differences in IL-2-induced activation in CD4^+^ Tregs and CD8^+^ Tregs can be correlated with twofold higher levels of CD25 basal expression in CD4^+^ Tregs compared with CD8^+^ Tregs (Figure 5B).

### *In vivo* sensitivity to IL-2 of CD4^+^ Tregs and CD8^+^ Tregs

**Figure 6:**
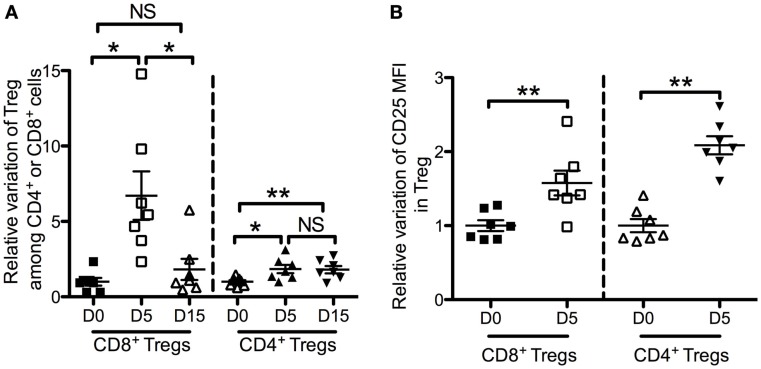
**Effects of low-dose IL-2 on Foxp3^+^ CD25^high^ CD127^low^ CD4^+^ T cells (CD4^+^ Tregs) and Foxp3^+^ CD25^high^ CD127^low^ CD8^+^ T cells (CD8^+^ Tregs) in humans**. Seven type 1 diabetes patients were treated with 3 MIU IL-2/day for 5 days (34). Time-course changes of CD8^+^ Tregs and CD4^+^ Tregs percentages in peripheral blood **(A)** and of CD25 mean fluorescence intensity (MFI) in CD8^+^ Tregs and CD4^+^ Tregs in peripheral blood **(B)** following a 5-days course of low-dose IL-2. Individual values and mean ± SEM are shown. Results are expressed in fold variation compared with the mean at day 0 of all the samples.

We next assessed the dynamics of CD4^+^ Tregs and CD8^+^ Tregs under low-dose IL-2 treatment, which is known to expand and activate CD4^+^ Tregs. Seven patients with type 1 diabetes received 3 MIU of IL-2/day for 5 days (34). This led to a significant increase of CD8^+^ Tregs, which reached 6.7 ± 4.3-fold at day 5 (Figure 6A). This CD8^+^ Treg expansion was not sustained at day 15, i.e., 10 days after the last IL-2 injection. IL-2 had a less pronounced effect on CD4^+^ Tregs, reaching a 1.8 ± 0.7-fold expansion at day 5. Nevertheless, and unlike CD8^+^ Tregs, this increase was sustained until day 15 (1.8 ± 0.6-fold). Interestingly, CD25 mean fluorescence intensity (MFI) was increased both on CD8^+^ Tregs and CD4^+^ Tregs after 5-day IL-2 treatment (Figure 6B). This CD25 increase was slightly higher for CD4^+^ Tregs than for CD8^+^ Tregs (2.1 ± 0.3 versus 1.6 ± 0.4; *p* = 0.005).

**Figure 7:**
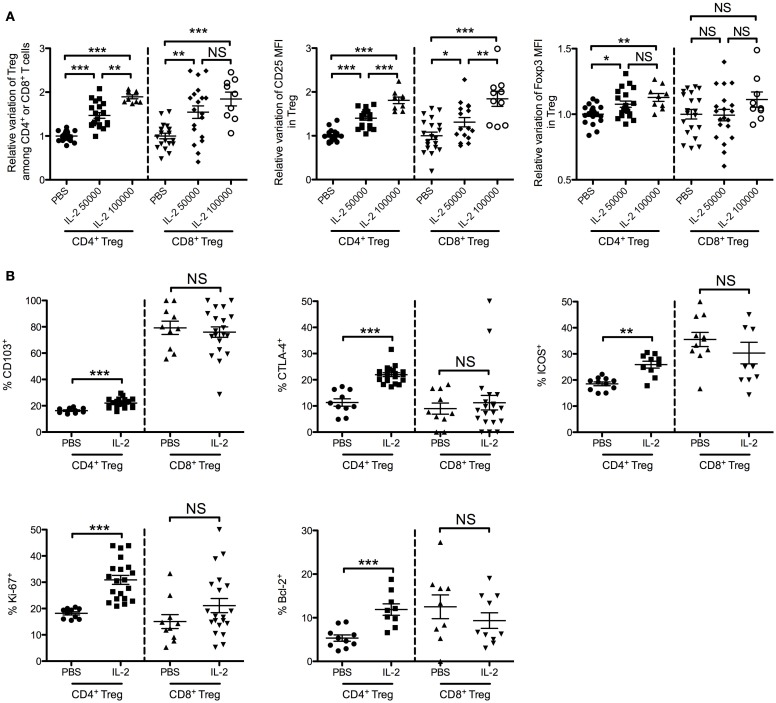
**Effects of low-dose IL-2 on CD4^+^ Tregs and CD8^+^ Tregs in mice**. Eight-week-old female BALB/c mice were injected daily for 5 days with PBS or with 50,000 or 100,000 IU of IL-2, and blood was sampled after the treatment. **(A)** Relative variations of percentages, CD25 MFI and FOXP3 MFI of CD4^+^ Tregs and CD8^+^ Tregs in blood after treatment (pool of three independent experiments). Individual values and mean ± SEM are shown. **(B)** Phenotypic activation markers of CD4^+^ Tregs and CD8^+^ Tregs after 5-day course of PBS (PBS) or 50,000 IU of IL-2 (IL-2) (pool of two independent experiments). Individual values and mean ± SEM are shown.

In BALB/c mice injected daily for 5 days with 50,000 or 100,000 IU of IL-2, we observed a similar dose-dependent increase of percentages of CD4^+^ and CD8^+^ Tregs in peripheral blood as compared with controls injected with PBS (Figure 7A). A 5-day course of 50,000 IU of IL-2 led to a 1.5 ± 0.3-fold increase of CD4^+^ Tregs and 1.5 ± 0.6-fold increase of CD8^+^ Tregs. At 100,000 IU/day, the increase was 1.9 ± 0.1-fold for CD4^+^ Tregs and 1.8 ± 0.5-fold for CD8^+^ Tregs. Similarly to the human study, we observed an IL-2 dose-dependent increase of CD25 expression in both CD4^+^ Tregs and CD8^+^ Tregs (Figure 7A). The fold increase of CD25 MFI induced by 50,000 or 100,000 IU of IL-2 was 1.4 ± 0.2 or 1.8 ± 0.2 in CD4^+^ Tregs and 1.3 ± 0.4 or 1.8 ± 0.5 in CD8^+^ Tregs, respectively. In contrast, FOXP3 expression levels were slightly increased only in CD4^+^ Tregs (1.1 ± 0.1 both with 50,000 and 100,000 IU of IL-2). No significant differences in FOXP3 expression level were observed in CD8^+^ Tregs at the two doses.

We also monitored some activation and proliferation markers in CD4^+^ Tregs and CD8^+^ Tregs after a 5-day course of 50,000 IU of IL-2. Interestingly, CD103, CTLA-4, ICOS, Ki-67, and Bcl-2 expression levels were increased after IL-2 treatment in CD4^+^ Tregs but not in CD8^+^ Tregs (Figure 7B and representative stainings in Figure S2 in Supplementary Material). These results highlight the specific biology of CD8^+^ Tregs compared with conventional CD4^+^ Tregs.

## Discussion and Conclusion

### On bona fide CD8^+^ Tregs

CD8^+^ Tsups are heterogeneous and often poorly characterized if not controversial cells in mice and humans. We focused our study on CD8^+^CD25^+^FOXP3^+^ T cells, which we call CD8^+^ Tregs, because they are identified based on the same markers as CD4^+^ Tregs. CD8^+^ Tregs are present at steady state at low levels both in human and mouse peripheral blood (about 0.4 and 0.1%, respectively). We previously reported lower percentages of CD8^+^ Tregs in blood of normal volunteers (0.22 ± 0.1%) (28), but we were using a different flow cytometry staining strategy. Because we optimized our flow cytometry analyses (choice of antibodies, analyses of whole blood with Perfix-NC kit), we believe that our new values accurately reflect their percentages. Natural CD8^+^CD25^+^FOXP3^+^ T cells are also present at very low frequencies (between 0.3 and 1.8% in CD8^+^ T cells) in blood, LNs, thymus, and spleen from naive non-human primates (37, 38).

We report here that CD8^+^ Tregs, as CD4^+^ Tregs, constitutively express CD25 and are sensitive to *in vitro* IL-2 activation, to which they respond by STAT5 phosphorylation. Like CD4^+^ Tregs, CD8^+^ Tregs also express markers associated with CD4^+^ Treg suppressive activity such as ICOS (39), CD103 (40, 41), and CTLA-4 (42, 43) at higher levels than other CD8^+^ T cells. It is of note that nearly 80% of CD8^+^ Tregs are CD103^+^, a marker associated with suppressive function of CD8^+^ T cells (21, 40, 44). This phenotype is associated with a high suppressive activity *in vitro* in classic functional assays. Similar suppressive activities of CD8^+^ Tregs have also been reported in humans (28) and non-human primates (45). Altogether, CD8^+^FOXP3^+^CD25^+^ T cells share phenotypic and functional characteristics with thymic CD4^+^ Tregs and are thus bona fide CD8^+^ Tregs, a designation that should be restricted to this population.

As for CD4^+^ Tregs, it seems that there could be a population of induced CD8^+^ Tregs. CD8^+^Foxp3^+^ can be induced *ex vivo* from CD8^+^Foxp3^−^ cells after culture in the presence of IL-2 and TGF-beta, but are only mildly suppressive (45, 46). Following simian immunodeficiency virus (SIV) infection, these peripheral CD8^+^CD25^+^Foxp3^+^ regulatory T cells are largely induced not only in blood but also in colorectal mucosa (37, 38). These cells have a regulatory phenotype (CD25^+^, CTLA-4^+^, CD28^+^, CD127^−^) (26, 28) with high proliferative capacities as 60% of induced CD8^+^ Tregs are Ki-67^+^. They express low levels of granzyme B and perforin, suggesting that their suppression is not mediated by killing.

### On differences between CD4^+^ Tregs and CD8^+^ Tregs

While CD8^+^ Tregs and CD4^+^ Tregs share functional suppressive activity, differences in their biology have been reported. IL-2 can bind the high-affinity trimeric IL-2 receptor (IL-2R) (CD25, CD122, and CD132) or the low-affinity dimeric IL-2R (CD122 and CD132) (47, 48). The trimeric IL-2R is constitutively expressed on CD4^+^ Tregs, but only transiently expressed on CD4^+^ and CD8^+^ T cells following T cell receptor (TCR) activation (47). We show here that CD8^+^ Tregs constitutively express the trimeric IL-2R, but compared with CD4^+^ Tregs, CD8^+^ Tregs express fewer CD25 molecules on their cell surface. CD4^+^ Tregs are two to fourfold more sensitive to IL-2 activation than CD8^+^ Tregs, although both CD4^+^ and CD8^+^ Tregs are equally 20 times more sensitive to IL-2 than their FOXP3^−^ counterparts. However, despite these differences in favor of CD4^+^ Tregs *in vitro*, we observed that the sensitivity of CD8^+^ Tregs to IL-2 *in vivo* is higher than that of CD4^+^ Tregs (Figure 6A), a so far unexplained observation. In agreement with our observations, a very large increase of CD8^+^ Tregs, greater than that for CD4^+^ Tregs, has been observed in blood of non-human primates following IL-2 treatment (49) in a context of tuberculosis infection.

Upon IL-2 activation, CD25 up-regulation is lower for CD8^+^ than for CD4^+^ Tregs. Also, after IL-2 treatment, activation markers such as CD103, CTLA-4, and ICOS, which are increased in CD4^+^ Tregs, are not increased in CD8^+^ Tregs, suggesting that IL-2 regulates CD8^+^ Tregs differently than CD4^+^ Tregs. Further studies are needed to clarify the molecular effects of IL-2 on CD8^+^ Tregs.

Importantly, we showed that CD8^+^ Tregs suppress *in vitro* effector T cell proliferation as well as or even better than CD4^+^ Tregs. This experiment has been performed only in mice as for now the low number of bona fide CD8^+^ Tregs in peripheral blood from human healthy donors does not allow to perform suppressive assays with these cells. However, we have already showed that non-manipulated human cord blood CD8^+^ Tregs are suppressive (50). It has also been reported that CD8^+^ Tregs from healthy non-human primates suppress as well as CD4^+^ Tregs (45). Thus, we can assume that, even if CD8^+^ Tregs are less numerous than CD4^+^ Tregs in peripheral blood of human healthy donors, their excellent suppressive activity makes them important cells to consider in the regulation of immune responses.

For now, it has previously shown by others and us that *in vivo* IL-2 expanded CD4^+^ Tregs have better *in vitro* suppressive activities than non-expanded CD4^+^ Tregs. It remains to evaluate whether this is also true for CD8^+^ Tregs functions.

### On the possible role of CD8^+^ Tregs

Several mechanisms have been implicated in the suppressive activity of suppressive CD8^+^ T cells, including direct lysis of target cells and secretion of immunosuppressive cytokines (14).

CTLA-4 has been shown to play a role in cell–cell contact-dependent mechanisms of CD4^+^ Tregs and CD8^+^ Treg-mediated suppression (51). As CD4^+^ Tregs, CD8^+^ Tregs may impede the up-regulation of CD80 and CD86 on immature dendritic cells or down-modulate them on mature dendritic cells (42) via CTLA-4, thereby inhibiting activation of Teffs. Jebbawi et al. (50) described a similar natural CD8^+^ Treg population from cord blood. This population expresses CTLA-4 and can secrete IL-10 and TGF-beta compared with CD8^+^CD25^−^ T cells.

CD8^+^ Tregs have also been implicated in the regulation of human AID, including inflammatory bowel disease (22). Eusebio et al. (52) showed also that asthma patients have fewer CD8^+^ Tregs in blood than healthy control subjects. Furthermore, FOXP3 mRNA levels of CD8^+^ T cells were significantly decreased in patients with severe asthma compared with mild to moderate asthma and control patients. Thus, natural CD8^+^ Tregs could play a major role in control of allergic inflammation.

In cancer, Chaput et al. (28) and Kiniwa et al. (53) demonstrated that CD8^+^ Tregs from colorectal and prostate tumors have strong immunosuppressive properties, and may contribute to tumoral immune escape and disease progression.

As many groups have reported the presence of CD8^+^ Tregs after induction either by a drug or by pathological conditions (AIDs, cancer, infection, allergy), it remains to be seen in these models whether they are induced by pathological or natural conditions. In any case, we demonstrate here that bona fide CD8^+^ Tregs are present at steady state in mice and humans.

### Conclusion

Many questions remain, especially about the origin and role of natural CD8^+^ Tregs in health and disease. Their striking phenotypic similarities with CD4^+^ Tregs, robust suppressive activity *in vitro* and exquisite sensitivity to IL-2 warrant further studies to fully appreciate their clinical relevance in AIDs and inflammatory diseases, and in IL-2 treatment efficacy.

## Conflict of Interest Statement

Guillaume Churlaud, Michelle Rosenzwajg, Bertrand Bellier, and David Klatzmann are shareholders of ILTOO pharma, a company with the exclusive license for a patent (of which Michelle Rosenzwajg and David Klatzmann are the inventors) owned by the authors’ academic institution and claiming the use of low-dose IL-2 in autoimmune diseases.

## Supplementary Material

The Supplementary Material for this article can be found online at http://journal.frontiersin.org/article/10.3389/fimmu.2015.00171
