# Supplementary material for: Human and Mouse CD8+CD25+FOXP3+ Regulatory T Cells at Steady State and during Interleukin-2 Therapy
Source: Front Immunol. 2015 Apr 15;6:171. doi: 10.3389/fimmu.2015.00171 (PMC4397865; doi:10.3389/fimmu.2015.00171)
Supplement: Supplementary file 1 [file Data_Sheet_1.PDF]

**Supplementary Material**

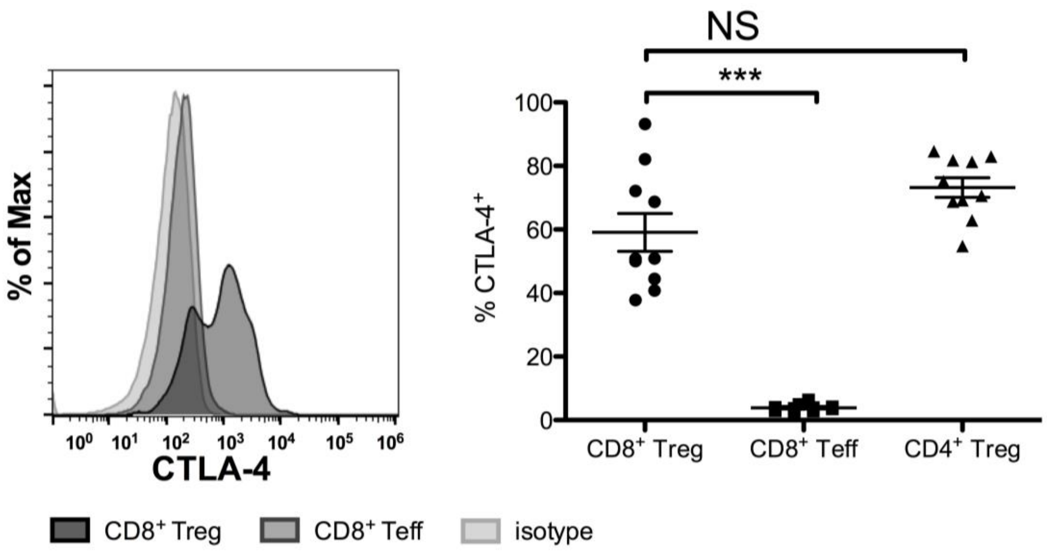


**Supplementary Figure 1: CTLA-4 characterization of CD8⁺CD25⁺FOXP3⁺ Tregs (CD8⁺ Tregs) compared with CD8⁺CD25⁻FOXP3⁻ effector CD8⁺ T cells (CD8⁺ Teffs) and with CD4⁺ Tregs in human healthy donors.** Representative histogram showing expression of CTLA-4 in CD8⁺ Teffs (gray) and in CD8⁺ Tregs (dark grey) compared to isotype control (light grey) are shown. Corresponding percentages in the overall population are shown. Individual values and mean ± SEM are shown.

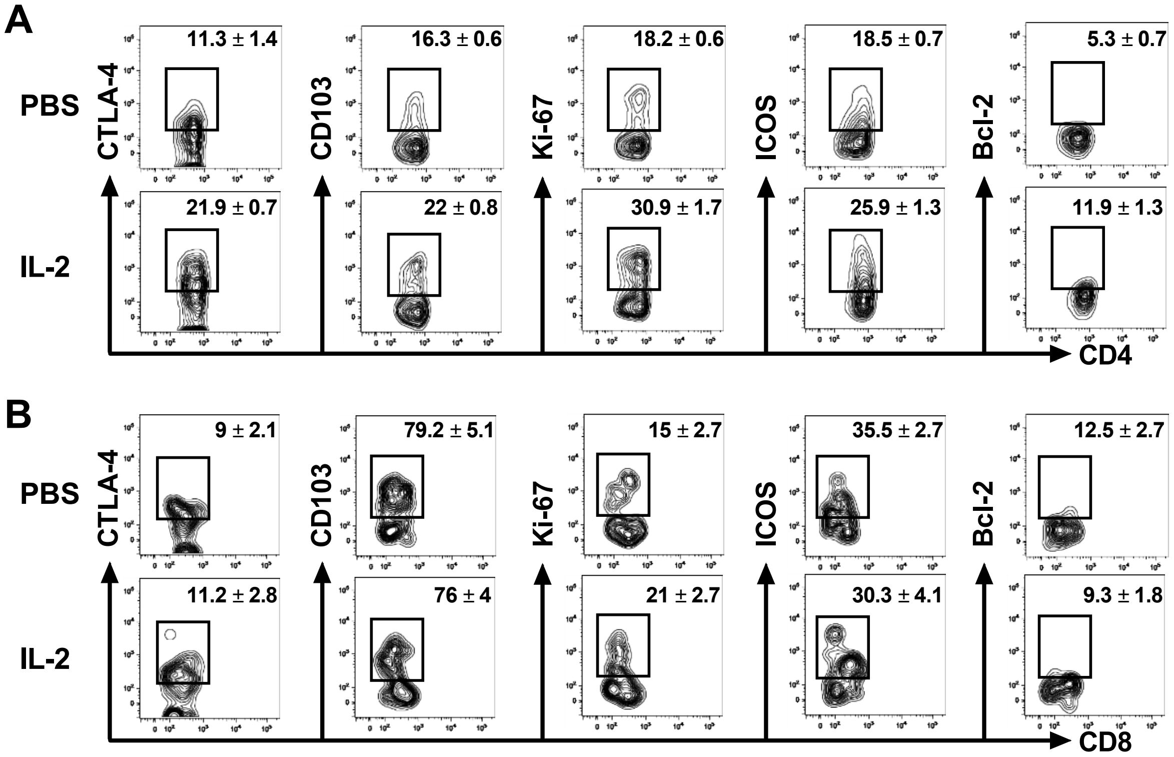


**Supplementary Figure 2: Effects of low-dose IL-2 on the expression of different markers on CD4⁺ Tregs and CD8⁺ Tregs in mice.** Eight-week- old female BALB/c mice were injected daily for 5 days with PBS or with 50,000 IU of IL-2. Flow cytometry representative expressions of CTLA-4, CD103, Ki-67, ICOS and Bcl-2 in $CD4^+$ Tregs (A) and $CD8^+$ Tregs (B) from peripheral blood after PBS or IL-2 treatment. $CD4^+$ Tregs were gated in $CD4^+$ cells among $CD3^+$ cells, and $CD8^+$ Tregs were gated in $CD8^+$ cells among $CD3^+$ cells. Representative means ± SEM are shown. Gates were set up using isotypic control.
